# Editorial: Multimodality Imaging in Acute Coronary Syndrome

**DOI:** 10.3389/fcvm.2022.939428

**Published:** 2022-05-27

**Authors:** Yining Wang, Zhao Wang, Jinwei Tian, Minjie Lu

**Affiliations:** ^1^Department of Magnetic Resonance Imaging, Fuwai Hospital, State Key Laboratory of Cardiovascular Disease, National Center for Cardiovascular Diseases, Chinese Academy of Medical Sciences and Peking Union Medical College, Beijing, China; ^2^School of Electronic Science and Engineering, University of Electronic Science and Technology of China, Chengdu, China; ^3^Department of Cardiology, The Second Affiliated Hospital of Harbin Medical University, Harbin, China; ^4^Laboratory of Cardiovascular Imaging (Cultivation), Chinese Academy of Medical Sciences, Beijing, China

**Keywords:** cardiac computed tomographic imaging, cardiac magnetic resonance imaging (CMR), echocardiography, multi-modality imaging, acute coronary syndrome (ACS)

Acute coronary syndrome (ACS) is a group of clinical syndromes caused by acute myocardial ischemia, mainly including ST-segment elevation myocardial infarction (STEMI), non-STEMI (NSTEMI), and unstable angina. It is commonly attributed to the rupture of atherosclerotic plaques and secondary acute intracoronary thrombosis, and patients may die from acute myocardial ischemia after experiencing chest discomfort and dyspnea (1). Each year, more than 7 million people are diagnosed with ACS worldwide (2). With such a large base population, timely diagnosis and intervention are crucial for saving lives. For clinicians, understanding the characteristics and capabilities of each imaging modality can help in the rapid selection of appropriate examination within the prime time window. Imaging is essential for providing the comprehensive assessment of the anatomy and physiology of coronary arteries in the diagnosis, monitoring, prognostic evaluation of ACS, and beyond to maximize patient benefit.

Selected papers in this research theme discuss recent advances in multi-modality imaging (MMI) of ACS including both non-invasive and invasive techniques, which provide great insight into the etiology and pathology of ACS, and also demonstrate application of new technologies to refine the existing diagnostic system for ACS.

## Echocardiography

As the preferred screening tool for patients with suspected coronary artery disease (CAD), stress echocardiography can increase diagnostic efficacy and reduce medically induced injury but still with unsatisfactory specificity and sensitivity (3). Lin et al. reported the enhanced effect of global myocardial work efficiency (GWE) as a novel parameter of left ventricular function measurement, affirming the improved diagnostic ability of GWE in combination with myocardial layer-specific strain for CAD.

Left ventricular systolic and diastolic dysfunction suggests possible structural myocardial injury, and the predictive role of a single left ventricular diastolic function (LVDF) marker for the occurrence of adverse events in patients with post-acute myocardial ischemia (AMI) is well-established. Bae et al. described how the sum of the abnormal diastolic function markers was measured by transthoracic echocardiography, from which the left ventricular wall motion score index (WMSI) was calculated. They emphasized the application of this comprehensive scoring method for long-term prognosis and risk stratification of CAD.

## Contrast Enhanced Cardiac Computed-Tomography

Approximately 6% of AMI patients do not exhibit acute coronary obstruction (4). There are also a large number of patients who are unable to undergo cardiovascular magnetic resonance (CMR) due to contraindications or equipment unavailability. Using a novel CT-based imaging approach, Ling et al. provided comprehensive assessment of coronary structure and function in the above population. With the add-on of late iodine enhancement (LIE) for myocardial scar identification, dynamic computed tomography myocardial perfusion imaging (CT-MPI) combined with coronary computed tomographic angiography (CCTA) is not only equivalent to CMR in etiologic assessment, but also provides additional visualization of coronary artery wall and luminal structures to guide clinical intervention and prognostic management of patients with ACS. CCTA assessment of functional coronary stenosis is challenging and requires myocardial blood flow (MBF) as a complement to physiological information, which is generally obtained by positron emission tomography (PET) and magnetic resonance imaging (MRI). However, CT-MPI, also as a MBF assessment tool, has limited use in clinical practice. The study by Lyu et al. obtained MBF cut-off values for dynamic CT-MPI which can differentiate healthy subjects from patients with functional myocardial ischemia, establishing the basis for the development of future universal datasets.

Unrecognized myocardial infarction (UMI) accounts for approximately more than 50% of all myocardial infarctions and is difficult to recognize due to its asymptomatic syndromes (5, 6). In this setting, late gadolinium enhancement (LGE) is able to detect unrecognized myocardial scar in a subset of STEMI patients, but has limited evidence in NSTEMI patients. Matsuda et al. demonstrated the ability of CCTA to detect non-infarct-related UMI before invasive coronary angiography.

Intravascular ultrasound (IVUS) or optical coherence tomography (OCT) enables high resolution imaging of atherosclerotic plaques but is limited by its invasive nature. With the rapid development of non-invasive imaging technologies, it would be interesting to see whether non-invasive methods can approach the diagnostic accuracy of invasive methods for plaque characterization. Luo et al. validated the ability of serum soluble suppression of tumorigenicity-2 (sST2) to predict plaque tissue composition in patients with NSTEMI using standard CCTA and coronary angiography. Assessment of coronary lesions by inflammatory factors combined with imaging techniques may be one of the key future directions.

The diagnostic value of CCTA on unstable plaques and surrounding environment was likewise confirmed by Lu et al. CCTA stood out when clinicians need an “one-stop shop” examination to assess coronary anatomy, histological features of atherosclerotic plaques and hemodynamics. It can comprehensively evaluate the condition of almost all coronary arteries and branches macroscopically, while identifying the characteristics of high-risk atherosclerotic plaques with high precision. The authors discussed the application of CCTA in predicting the occurrence of ACS and patient risk assessment, and provided an overview of some emerging techniques such as computational fluid dynamics.

The predictive function of coronary calcification on cardiovascular morbidity and mortality has been confirmed by previous studies and CT is considered the gold standard. Zhang et al. enumerated the diagnostic performance of different types of CT techniques in the assessment of vascular calcification including coronary calcification. Multi-slice spiral CT and dual-energy CT are commonly used in clinical practice to detect and quantify the extent of arterial calcification and assess high-risk plaque features. Emerging CT techniques including multi-slice spinal CT, micro CT, ultra-high resolution CT and etc., aim to enhance the detection of microcalcification by improving the imaging resolution.

The Coronavirus Disease 2019 (COVID 19) pandemic challenges traditional ACS assessment pathways, and it is sometimes of high demand to limit the spread of infection by avoiding unnecessary invasive procedures and curtailing the length of patient stay. Based on the GRACE Score, hemodynamics, and patients' COVID19 status, Alasnag et al. established the ACS cardiac computed tomography (CCT) protocol and treatment strategy, and validated the role of CCT in the evaluation of low risk ACS. Although this study is only a single-center trial, it has demonstrated the role of CCT in the anatomical risk stratification and has encouraged us to take on new challenges in special settings when working with conventional imaging.

## Cardiac Magnetic Resonance Imaging (CMR)

As LGE has become an established technique for marking infarcted myocardium *in vivo*, CMR can be used for risk stratification and prognostic management of patients with STEMI (7, 8). Subtle and localized myocardial dysfunction can be further detected by strain analysis, but the correlation of cardiac MRI signature tracing with conventional CMR parameters is not yet known. Yu et al. demonstrated that strain not only correlates closely with LGE performance, but also detects alterations in myocardial function that are missed by left ventricular ejection fraction (LVEF) assessment and myocardial enhancement.

Left ventricular remodeling after STEMI is associated with adverse cardiovascular events in patients, and current guidelines recommend that patients improve their prognosis by taking aspirin prior to percutaneous coronary intervention (PCI) (7, 9). Calvieri et al. demonstrated the role of CMR in assessing the efficacy of cardioprotective therapies. CMR feature tracking analysis (CMR-FT), which is able to quantitatively assess subtle deformation in response to myocardial motion, detected adverse ventricular remodeling at an early stage.

Impairment of LV strain predicts possible subsequent ventricular remodeling and worsening ejection fraction, raising concerns among clinicians. With the development of new therapies, short-term mortality in patients with STEMI has decreased substantially, however, long-term prognosis varies widely. Accurate and clinical available indicators for risk stratification are able to aid in the early identification of high-risk patients. Lai et al. affirmed the established role of CMR myocardial strain measurements for adverse events and the prognostic significance of the combined assessment of both left and right ventricular strain indexes.

## Cardiac Nuclear Medicine

Complications of diabetes often involve the microcirculatory system, and patients can develop angina secondary to microcirculatory dysfunction. Qi et al. reviewed the progress of commonly used imaging methods in detecting coronary microvascular angina. Both single-photon emission computed tomography (SPECT) and PET-MPI quantify myocardial blood flow, with the latter being more sensitive and specific. CMR is sensitive to early lesions and allows qualitative and quantitative visualization of coronary microcirculation. Myocardial contrast echocardiography is commonly used for preliminary screening and in recent years has also been developed for therapeutic purposes. CT allows for a comprehensive assessment of non-coronary ischemic heart disease (INOCA).

## IVUS, OCT, FFR, and QFR

Kubo et al. evaluated the combined use of multiple intravascular imaging techniques in ACS with their respective strengths and weaknesses. Both IVUS and OCT provide cross-sectional images of coronary arteries. IVUS provides better penetration, and can assess the size of both the lumen and external elastic lamina of the vessel, and therefore is valuable for assessing plaque burden. OCT has higher resolution but shallower penetration depth, and can detect thin cap fibroatheroma, also known as vulnerable plaque (10, 11), which has a large necrotic core and a thin fibrous cap often with macrophage infiltration. OCT can differentiate all major types of atherosclerotic plaques including lipid, calcification, fibrous plaques and etc. Near-infrared spectroscopy (NIRS) can detect the lipid component of plaques. They emphasized that any single technique has limitations in assessing the pathology of ACS and that a combination of multi-modality imaging techniques is needed to perform a comprehensive assessment and guide lesion-specific treatment. Usui et al. demonstrated that the examination results between OCT and NIRS are correlated, offering the possibility of assessing the morphological and molecular characteristics of lipid-rich plaques by a single system.

Cao et al. used OCT to evaluate the predictors of erosion-prone plaques, and found that some atherosclerotic plaques with certain features and geometry are more prone to develop secondary plaque erosion, which is one of the most common causes of coronary thrombosis.

Several studies have shown that there are anatomical, pathological and clinical differences between male and female CAD patients, and using the same cardiovascular assessment criteria does not provide appropriate treatment recommendations and prognostic assessments. Hou et al. clarified the gender difference of quantitative flow ratio (QFR) in patients with STEMI, providing evidence for future establishment of a personalized treatment system. They focused on patients with poorer prognosis for non-infarct-related arterial disease (NIRA), who may require a more aggressive treatment approach.

Previous studies have shown that both the immune system and inflammatory cells promote plaque progression and aggravate coronary artery stenosis (12, 13). Liu C. et al. demonstrated that immunoinflammatory biomarkers such as interleukin (IL)-6, tumor necrosis factor (TNF)-α, and interferon (IFN)-γ were associated with a high risk of QFR ≤ 0.8. It is suggested that combining inflammatory markers with imaging parameters can significantly improve the identification of functional severe CAD by predicting functional ischemia and anatomic stenosis.

PCI is the one of the routine treatments for STEMI, but approximately 20% patients who fail functional revascularization still experience adverse coronary events after the procedure. FFR can predict patient prognosis by evaluating functional stenosis of the coronary arteries. Wang et al. found that coronary physiology and deceleration capacity (DC) combined with QFR were able to improve the predictive accuracy of major adverse cardiac and cerebrovascular events.

## Artificial Intelligence (AI)

The comprehensive assessment of ACS often involves multiple imaging modalities, demanding real-time, objective and precise diagnosis. Liu M-h. et al. discussed the advantages of AI combined with MMI in filling the gaps of ACS management strategies. With massive data collection and powerful processing capabilities, AI, in particular deep learning, learns and exacts hierarchal features from clinical data automatically and is valuable for lesion identification and segmentation, disease classification, clinical recommendation, and prognosis prediction. With the help of AI, diagnostic performance can be significantly improved ranging from non-invasive imaging techniques such as CT and CMR to invasive imaging techniques such as coronary angiography (CAG), OCT and IVUS.

## Conclusion and Future Perspectives

It's thrilling to see the fast pace multimodality imaging technologies have been developed in the evaluation of ACS for the past decade. In addition, the advances of new technologies can further help physicians and bring benefits to patients. The combination of multiple imaging modalities, the addition of AI technology and the joint application of biological markers and imaging techniques have already evolved into mainstream directions for future research, which may bring fundamental changes to the diagnosis and management of ACS.

## Author Contributions

YW wrote the first draft of the manuscript. ZW, JT, and ML wrote sections of the manuscript. All authors contributed to manuscript revision, read, and approved the submitted version.

## Funding

This work was supported by the Construction Research Project of the Key Laboratory (Cultivation) of Chinese Academy of Medical Sciences (2019PT310025), National Natural Science Foundation of China (81971588), Youth Key Program of High-level Hospital Clinical Research (2022-GSP-QZ-5), the Applied Technology Research and Development Plan of Heilongjiang Province (GY2020YF0039 to JT) and the Fok Ying-Tong Education Foundation for Young Teachers (171032 to JT).

## Conflict of Interest

The authors declare that the research was conducted in the absence of any commercial or financial relationships that could be construed as a potential conflict of interest.

## Publisher's Note

All claims expressed in this article are solely those of the authors and do not necessarily represent those of their affiliated organizations, or those of the publisher, the editors and the reviewers. Any product that may be evaluated in this article, or claim that may be made by its manufacturer, is not guaranteed or endorsed by the publisher.
